# The epidemiology of bloodstream infection contributing to mortality: the difference between community-acquired, healthcare-associated, and hospital-acquired infections

**DOI:** 10.1186/s12879-022-07267-9

**Published:** 2022-04-05

**Authors:** Seok Jun Mun, Si-Ho Kim, Hyoung-Tae Kim, Chisook Moon, Yu Mi Wi

**Affiliations:** 1 Division of Infectious Diseases, Department of Internal Medicine, Inje University College of Medicine, Inje University Busan Paik Hospital, Busan, South Korea; 2 Division of Infectious Diseases, Samsung Changwon Hospital, Sungkyunkwan University School of Medicine, 158, Paryong-ro, Masanhoewon-gu, Changwon, Gyeongsangnam-do 51353 Republic of Korea; 3 Department of Laboratory Medicine, Samsung Changwon Hospital, Sungkyunkwan University School of Medicine, Changwon, Republic of Korea

**Keywords:** Bloodstream infection, Mortality, Hospital-acquired infection, Antimicrobial resistance

## Abstract

**Background:**

The epidemiology of bloodstream infection (BSI) is well-established; however, little is known about the contribution of different pathogens to mortality. To understand true burden of BSI, pathogens contributing to mortality were investigated and compared according to where the BSI was acquired.

**Methods:**

Data from deceased patients in two teaching hospitals in the Republic of Korea were collected. BSI contributing mortality was defined as BSI within 2-weeks before death. Cases were grouped by acquisition sites: community-acquired (CA)-, healthcare-associated (HCA)-, and hospital-acquired (HA)-BSI. Drug resistance, BSI focus, and appropriateness of empirical antimicrobial therapy were also compared.

**Results:**

Among 1849 deceased patients in the hospitals, 280 (15.1%) patients experienced BSI within 2-weeks before death. In all, 71, 53, and 156 patients in the CA-, HCA-, and HA-BSI groups, respectively, with 316 total isolated pathogens were analyzed. The three most common pathogens were *Klebsiella pneumoniae* (17.1%)*, Escherichia coli* (16.4%), and *Staphylococcus aureus* (11.4%). While *K. pneumoniae* and *E. coli* were the most common pathogens in CA- and HCA-BSI, *Acinetobacter baumannii* and *Candida* species were in HA-BSI. 26.3% (41/156) of patients experienced breakthrough HCA-BSI during administration of carbapenem and/or vancomycin. The proportion of central venous catheter-related infection (0%, 3.4% and 28.3%), carbapenem resistant-Gram negative bacilli (0%, 6.9% and 21.9%), and inappropriate empirical antimicrobial therapy (21.1%, 37.7% and 51.9%; all P < 0.001) were more frequently observed in HA-BSI.

**Conclusion:**

The epidemiology of BSI related to mortality had unique characteristics according to the acquisition site. Given the epidemiology of HA-BSI, infection control and antibiotics stewardship programs should be emphasized.

**Supplementary Information:**

The online version contains supplementary material available at 10.1186/s12879-022-07267-9.

## Background

Bloodstream infections (BSI) are a common cause of mortality despite advances in antimicrobial agents and sepsis management [1–3]. Although short-term mortality of BSI has been estimated from 12 to 34%, the range of mortality could be highly variable according to the acquisition site (whether community or hospital-acquired BSI), a specific population, pathogens, drug resistance, and geographical region [1–8]. For example, hospital-acquired (HA)-BSI usually show higher crude mortality than community-acquired (CA)-BSI [2]. Although *Escherichia coli* is the most common pathogen causing BSIs worldwide, higher mortality has been reported with bacteremia due to other Gram-negative bacteremia other than *E. coli* [3, 9, 10]. Therefore, the epidemiology of pathogens contributing to mortality may differ from the actual bacteremia incidence of each specific pathogen.

In the Republic of Korea, *E. coli*, *Staphylococcus aureus*, and *Klebsiella pneumoniae* were the three most common pathogens of true bloodstream infection; In reports after the 2000s, drug resistance of Gram-negative bacilli (GNB) has been a more predominant problem than that of Gram-positive cocci (GPC) [10, 11]. Carbapenem resistance has been frequently observed with *Acinetobacter baumannii*, and reported to be more than 90% in a surveillance study in 2017 [12]. Although there are data on the proportion of each pathogen isolated from BSIs and their drug resistance, data on pathogens causing mortality and the extent of their contribution are rarely reported.

We believed that knowing the epidemiology of BSIs contributing to mortality and their susceptibility patterns could help better understand BSIs and set priority for infection control and institutional or national antibiotic policy. Therefore, this retrospective study was conducted to investigate pathogens related to mortality and to compare species and their characteristics according to where the BSI was acquired.

## Methods

### Study design and definition

Adult patients (≥ 18 years old) admitted to one of two teaching hospital between January 2019 and December 2019 were enrolled. The participating institutions were Samsung Changwon Hospital and Inje University Busan Paik Hospital located in Changwon and Busan, Korea, respectively. Electronic medical records were reviewed to collect data of laboratory results and clinical characteristics of patients.

We defined BSIs contributing mortality as a BSI within the 2-weeks before death, as prior studies have used a 14-day mortality to represent short-term mortality from BSI [8]. The results of blood cultures performed on the study population during the 2-weeks before death were collected. When bacteria or fungi were isolated from more than two separate blood samples or from a single blood sample in patients with explainable clinical symptoms and focuses other than skin commensals, they were considered as true BSIs [13]. We only considered isolated pathogens from true BSI as the pathogens contributing to mortality.

Patients and their isolated pathogens were classified by their acquisition sites: Community-acquired (CA)-, healthcare-associated (HCA)- and HA-BSI. HCA infection was defined as infected patients with at least one of the four elements: (1) Parenteral treatment within 30 days, (2) outpatient chemotherapy or hemodialysis within 30 days, (3) hospitalization for ≥ 2 days in the preceding 90 days and (4) nursing home residence [14]. To compare baseline characteristics of patients and pathogens in CA-, HCA-, and HA-BSI groups, the following data were collected: age, sex, underlying disease, prior major surgery (within 4 weeks), prior antibiotics use (within 4 weeks), infection focus of BSI, and appropriateness of empirical antimicrobial treatment. Appropriate empirical antibiotic use was defined when agents to which the microbes were susceptible were administered within 48 h after obtaining the blood culture sample. In addition, the antimicrobial susceptibility of each pathogen was classified into four major resistance patterns: cefotaxime-resistant *Enterobacteriaceae* (not including carbapenem-resistant), carbapenem-resistant GNB (CRGNB), methicillin-resistant *S. aureus* (MRSA), and vancomycin-resistant *Enterococcus* (VRE). *Stenotrophomonas maltophilia*, which has intrinsic resistance to carbapenem, was also classified as CRGNB [15]. In addition, difficult-to-treat resistance (DTR), defined as nonsusceptibility to all β-lactams and fluoroquinolones tested, was identified among CRGNB [16, 17].

### Blood culture and drug sensitivity test

Blood was taken via a peripheral vein and/or a central line. Blood was drawn for two sets of blood culture bottles (aerobic and anaerobic, 8-10 cc blood for each bottle), and culture bottles were incubated in the Bactec-9240 system (Becton Dickinson, Sparks, MD) or BacT/Alert 3D system (bioMérieux Inc., Marcy l’Etoile, France).). All samples were cultured in blood agar and MacConkey agar plates in a 35 °C incubator for 24 h and identified using the Vitek MS system (BioMérieux, Hazelwood, MI, USA). All antimicrobial susceptibility tests (ASTs) were performed using A Vitek II automated system (bioMérieux Inc.) according to the Clinical and Laboratory Standards Institute 2018 guidelines [18]. All procedures were performed according to the manufacturer’s instructions.

### Statistical analysis

All statistical analyses were performed using SPSS 23.0 for Window (IBM Corp., 2015, Chicago, IL, USA). To compare characteristics between the CA-, HCA-, and HA-BSI groups, a Student’s *t*-test or Mann–Whitney test was used to compare continuous variables of two groups, and a one-way analysis of variance (ANOVA) or Kruskal–Wallis test was used to compare continuous variables of multiple groups. Categorical variables were compared by using the chi-square test or Fisher’s exact test. For post hoc analysis for multiple comparison, the Bonferroni method was used. All P values were two-tailed, and P values < 0.05 were considered statistically significant.

## Results

### Study population

During the study period, a total of 1849 patients died. There were 331 patients with positive blood culture during the 2-weeks prior to death. After excluding two patients under 18 years of age and 49 patients with contaminated blood cultures, 280 (15.1%) patients were included in this study. The mean age of the study population was 69.92 years, and the ratio of males to females was 3:2. Seventy-one (25.4%), 53 (18.9%), and 156 (55.7%) patients were classified as CA-, HCA- and HA-BSI acquired infection, respectively. Whereas liver disease was more frequently observed in the CA-BSI group, cardiovascular disease was more frequently observed in the HA-BSI group. Malignancy and prior antibiotic use were more frequently observed in HCA- and HA-BSI groups than in the CA-BSI group (Table 1).

**Table 1:** Clinical characteristics of patients with BSI within 2-weeks before death

	Overall (280)	Community-acquired (71)	Healthcare-associated (53)	Hospital-acquired (156)	P value	Post-hoc analysis
Age	69.92 ± 12.95	72.99 ± 13.57	68.75 ± 12.37	68.92 ± 12.72	0.069	
Sex (male)	168 (60.0)	48 (67.6)	34 (64.2)	86 (55.1)	0.162	
Malignancy	118 (42.1)	19 (26.8)	34 (64.2)	65 (41.7)	< 0.001	HCA>CA,HA
Diabetes	85 (30.4)	24 (33.8)	13 (24.5)	48 (30.8)	0.532	
Hypertension	120 (42.9)	30 (42.3)	27 (50.9)	63 (40.4)	0.411	
Cardiovascular disease	73 (26.1)	13 (18.3)	8 (15.1)	52 (33.3)	0.007	HA>HCA
Pulmonary disease	26 (9.3)	7 (9.9)	1 (1.9)	18 (11.5)	0.093	
Liver disease	56 (20.0)	24 (33.8)	10 (18.9)	22 (14.2)	0.004	CA>HA
Rheumatologic disease	12 (4.3)	1 (1.4)	1 (1.9)	12 (4.3)	0.184	
Renal disease	25 (8.9)	3 (4.2)	3 (5.7)	19 (12.2)	0.136	
Solid organ transplantation	4 (1.4)	0 (0.0)	0 (0.0)	4 (2.6)	0.392	
Prior major surgery	35 (12.5)	0 (0.0)	4 (7.5)	31 (19.9)	< 0.001	HA>CA
Prior antibiotics use	124 (44.3)	2 (2.8)	34 (64.2)	88 (56.4)	< 0.001	HCA,HA>CA

Data represent the number (%) of patients, unless otherwise specified

*BSI* bloodstream infection; *HCA* healthcare-associated infection; *CA* community-acquired infection; *HA* hospital-acquired infection

### Species of isolated pathogens

A total of 316 pathogens were isolated from blood culture, and 74 (23.4%), 58 (18.4%) and 184 (58.2%) pathogens were identified from the CA-, HCA- and HA-BSI groups, respectively. Of the 280 patients, 34 patients experienced polymicrobial BSI in single BSI event or more than 2 consecutive BSI events, of which the HA-BSI group accounted for ~ 75% (26/34).

The five most common pathogens were *Klebsiella pneumonia* (17.1%)*, Escherichia coli* (16.4%), *Staphylococcus aureus* (11.4%), *Acinetobacter baumannii* (10.8%), and *Candida* species (9.2%). The commonest pathogen in CA-, HCA-, and HA-BSI groups were *E. coli*, *K. pneumoniae* and *A. baumannii* (Table 2). Common isolated pathogens from each hospital were relatively similar and are described in Additional file 1: Tables S1 and S2. No difference in patient age was observed among the pathogen groups (Additional file 1: Table S3).

**Table 2:** Isolated pathogens from bloodstream infections

Overall (316)	Community acquired (74)	Healthcare associated (58)	Hospital acquired (184)
*Klebsiella pneumonia* (54)	*Escherichia coli* (20)	*Klebsiella pneumonia* (17)	*Acinetobacter baumannii* (33)
*Escherichia coli* (52)	*Klebsiella pneumonia* (15)	*Escherichia coli* (11)	*Candida* spp. (28)
*Staphylococcus aureus* (41)	*Staphylococcus aureus* (14)	*Staphylococcus aureus* (7)	*Klebsiella pneumonia* (22)
*Acinetobacter baumannii* (34)	Other *Streptococcus* spp*.* (11)	*Pseudomonas aeruginosa* (4)	*Escherichia coli* (21)
*Candida* spp. (29)	*Proteus* spp*.* (3)	Other *Streptococcus* spp. (4)	*Staphylococcus aureus* (20)
*Enterococcus faecium* (24)	Others (11)*	*Enterococcus faecium* (3)	*Enterococcus faecium* (20)
Other *Streptococcus* spp. (21)		Others (12)*	*Pseudomonas aeruginosa* (9)
*Pseudomonas aeruginosa* (13)			*Stenotrophomonas maltophilia* (5)
*Enterococcus faecalis* (6)			Other *Streptococcus* spp. (5)
*Stenotrophomonas maltophilia* (6)			*Enterococcus faecalis* (3)
*Proteus* spp. (6)			*Coagulase (-) Staphylococcus* (3)
*Coagulase (-) Staphylococcus* (4)			Others (15)*
*Citrobacter* spp. (3)			
Others* (25)			

*Pathogens isolated from blood culture fewer than three times

Overall, 54.5% (85/156) of patients in the HA-BSI group had a central venous catheter, and 37.2% (58/156) of patients were admitted to the intensive care unit when BSI occurred (not shown in Table). In addition, 26.3% (41/156) of patients experienced breakthrough HCA-BSI during administration of carbapenem and/or vancomycin. The most commonly isolated pathogen during carbapenem therapy was *A. baumannii* (Additional file 1: Table S4).

### Comparison of characteristics between CA-, HCA- and HA-BSI

**Fig. 1:**
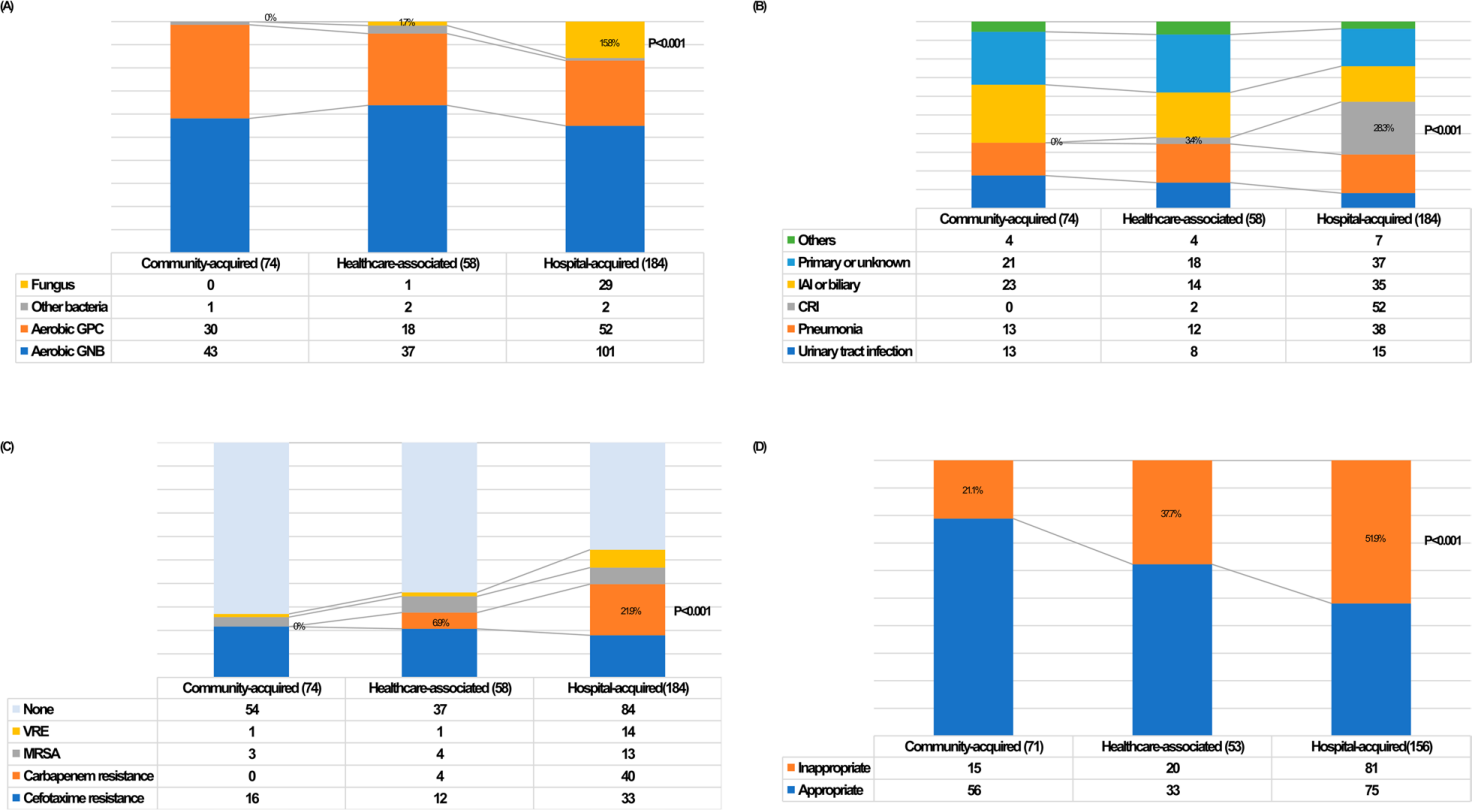
The comparison between community-acquired, healthcare-associated, and hospital-acquired bloodstream infection contributing to mortality. **A** Isolated pathogens, **B** Portal of entry, **C** Drug resistance, **D** Appropriateness of empirical treatment. *GPC* Gram-positive coccus; *GNB* Gram-negative bacillus; *IAI* intra-abdominal infection; *CRI* catheter-related infection; *VRE* Vancomycin-resistant *Enterococcus*; *MRSA* Methicillin-resistant *Staphylococcus aureus*

Figure 1 shows the characteristics of each BSI group. Almost all of the fungi were isolated from the HA-BSI group. *Candida* species comprised 96.6% (28/29) of fungi isolates, and *C. albicans* was the most predominant (17 cases of 28 *Candida* species) (Fig. 1A). Meanwhile, the most common portal of BSI entry was primary or from an unknown focus. Central venous catheter-related infection was more frequently observed in the HA-BSI group (28.3%) than in the HCA-BSI group (3.4%) or the CA-BSI group (0%) (Fig. 1B).

The most frequently observed drug resistance was cefotaxime-resistant *Enterobacteriaceae*, which was distributed relatively evenly amongst in the CA-BSI (21.6%), HCA-BSI (20.6%), and HA-BSI groups (17.9%). There was no significant difference in the VRE and MRSA proportions in the three BSI groups. However, CR-GNB was the predominant drug resistant pathogen in the HA-BSI group (21.9%) (Fig. 1C). The DTR pattern was identified in 81.8% (36/44) of CRGNBs. The most common species of CRGNB was *A. baumannii* (24 isolates), which rarely or never contributed to mortality in HCA- (6.9%) or CA-BSIs (0%). Inappropriate empirical antimicrobial treatment was more frequently observed in HA-BSIs (51.9%) compared with HCA- (37.7%) and CA-BSIs (21.1%) (Fig. 1D). In our study population, 65 cases of BSI were empirically treated with type 2 carbapenems and glycopeptides. However, the treatment was still inappropriate in 26.2% of these patients due to drug resistance (data not shown).

## Discussion

Our study showed common isolates from BSIs that contributed to mortality and patient clinical characteristics. Pathogens and characteristics of HA-BSIs related to mortality showed very different epidemiology from that of CA-BSIs in terms of species, portal of entry, antimicrobial resistance pattern, and appropriateness of empirical antimicrobial therapy. HA-BSIs contribute considerably to mortality in hospital, and the combination of carbapenem and glycopeptides—which might be the most preferred empirical antibiotics regimens of clinicians—did not prevent HA-BSIs nor did it meet the criterion of appropriate antimicrobial therapy.

GNBs more contributed to mortality than GPCs. Increasing antimicrobial resistance of GNB has been continuously reported and, along with the lack of new licensed antibiotics since the 1990s, has become a significant problem [10, 11, 19]. In this study, cefotaxime-resistant *Enterobacteriaceae* demonstrated the most predominant resistant pattern in the study population, especially in the CA-BSI group. Although ESBL-producing *Enterobacteriaceae* are usually associated with HA infection, its acquisition in the community setting has increased since the mid-2000s [20]. Surveillance data of antimicrobial resistance in South Korea in 2011 showed that only 18.3% of *E. coli* were resistant to cefotaxime; however, resistance rates increased to 34.7% by 2017 [10, 11]. Our study supported the real-world relationship between increasing ESBL-producing *Enterobacteriaceae* and mortality.

CRGNBs or DTR-GNBs has been evolving as a challenging health problem in hospital acquired infection [11, 16]. Numerically, 8.4% (156/1849) of patients who died in hospital had HA-BSI, and 25% (40/156) of these patients died because of acquisition of CRGNB bacteremia, which was most frequently caused by *A. baumannii*. Indeed, the high carbapenem resistance of *A. baumannii* has been reported worldwide [12, 21]. Although colistin is usually the only treatment option for carbapenem-resistant *Acinetobacter baumannii* (CRAB), the efficacy of colistin for CRAB bacteremia is not very reliable [22]. One multicenter study from South Korea reported the 28-day mortality of patients with CRAB bacteremia treated with colistin at 61.4% [23]. However, even for patients with CRAB bacteremia who did not receive appropriate antibiotics, the mortality rate was 69.8% [24]. High mortality, difficulty in administration of appropriate empirical antibiotics, and lack of reliable antimicrobial therapeutic options might be related to *A. baumannii* being the most predominant species from HA-BSI contributing to mortality in this study. Before effective treatment options against CRGNB to introduce, efforts are needed to comply with infection prevention management and to audit antibiotic prescriptions [25, 26].

In our study, *Candida* species were reported as the second most common isolates for HA-BSIs causing patient mortality. Appropriate empirical treatment of candidemia is another challenging problem similar to bacteremia caused by multidrug resistant pathogens. Although early appropriate treatment is associated with favorable outcomes [27], only a few patients received appropriate empirical anti-fungal agents due to difficulty in early diagnosis [28]. In our study, only 28.6% of patients who died with candidemia received appropriate antifungal agents. To reduce mortality resulting from candidemia, a more rapid and reliable diagnostic tool than blood culture is needed. The new T2Candida molecular test using magnetic resonance with molecular diagnostics has been introduced and is expected to be a rapid, promising tool for the detection of candidemia [29]. However, due to high initial and maintenance costs, there is a difficulty in wide application of T2 Candida in the clinical setting [30]. More accessible tools with high accuracy for candidemia diagnosis should be developed.

There were some limitations in our study. Even though our data were collected from two teaching hospitals, the epidemiology of BSI depends both on patient demographics and the geographical characteristics of each hospital. For example, only a small number of transplant recipients were included in our study; medical centers specializing in transplantation might show different HA-BSI epidemiology. In addition, in our study, we only considered short-term mortality of BSI. From this study, we cannot infer long-term mortality or changes in disability caused by BSI. Further studies are needed.

## Conclusions

In conclusion, GNBs comprised a larger portion of microbes related to mortality than GPCs. *K. pneumoniae* and *E. coli* were the predominate pathogens associated with mortality in CA- and HCA-BSIs, while *A. baumannii* and *Candida* species were for HA-BSI. Along with increasing drug resistance in GNB, inappropriate empirical therapy was primarily observed in the HA-BSI group because of difficulties in predicting drug resistance and identifying fungal pathogens. Our data showed that, in the hospital setting, a significant number of patients die from acquired BSI, which the infection control team should make every effort to prevent. A combination of carbapenem and glycopeptide led to other pathogens arising with higher resistance or to fungal infection. The importance of infection prevention and antibiotics stewardship program should be emphasized, especially until more reliable treatment options for multidrug resistant GNB and rapid diagnostic tools are introduced.

## Supplementary Information

**Additional file 1: Table S1.** Isolated pathogen from Samsung Changwon medical center. **Table S2.** Isolated pathogen from Inje University Busan Paik Hospital. **Table S3.** Age distribution of patients grouped by blood isolates (P = 0.838). **Table S4.** Breakthrough bloodstream infection in patients with hospital-acquired bloodstream infection.

## Data Availability

The datasets generated and/or analysed during the current study are not publicly available, but are available from the corresponding author on reasonable request.

## Abbreviations

BSI: Bloodstrean infection
CA: Community-acquired
CRAB: Carbapenem-resistant *Acinetobacter baumannii*
CRGNB: Carbapenem-resistant Gram-negative bacilli
DTR: Difficult-to-treat resistance
HA: Hospital-acquired
HCA: Healthcare-associated
GNB: Gram-negative bacilli
GPC: Gram-positive cocci
MRSA: Methicillin-*resistant Staphylococcus aureus*
VRE: Vancomycin-resistant *Enterococcus*
