# Supplementary material for: The epidemiology of bloodstream infection contributing to mortality: the difference between community-acquired, healthcare-associated, and hospital-acquired infections
Source: BMC Infect Dis. 2022 Apr 5;22:336. doi: 10.1186/s12879-022-07267-9 (PMC8981700; doi:10.1186/s12879-022-07267-9)
Supplement: Supplementary file 1 — Additional file 1: Table S1. Isolated pathogen from Samsung Changwon medical center. Table S2. Isolated pathogen from Inje University Busan Paik Hospital. Table S3. Age distribution of patients grouped by blood isolates (P = 0.838). Table S4. Breakthrough bloodstream infection in patients with hospital-acquired bloodstream infection. [file 12879_2022_7267_MOESM1_ESM.docx]

**Additional Table S1. Isolated pathogen from Samsung Changwon medical center**

| Overall (171) | Community-acquired (30) | Healthcare -associated (29) | Hospital-acquired (112) |
| --- | --- | --- | --- |
| *Klebsiella pneumonia* (29) | *Escherichia coli* (11) | *Klebsiella pneumonia* (10) | *Acinetobacter baumannii* (20) |
| *Escherichia coli* (24) | *Klebsiella pneumonia* (7) | *Escherichia coli* (5) | *Candida* spp. (20) |
| *Staphylococcus aureus* (24) | *Staphylococcus aureus* (5) | *Staphylococcus aureus* (4) | *Staphylococcus aureus* (15) |
| *Candida* spp. (21) | Other *streptococcus* spp. (3) | *Enterococcus faecalis* (2) | *Klebsiella pneumonia* (12) |
| *Acinetobacter baumannii* (20) | *Enterococcus faecalis* (1) | *Enterococcus faecium* (2) | *Enterococcus faecium* (12) |
| *Enterococcus faecium* (14) | *Enterococcus faecium* (1) | *Candida* spp. (1) | *Escherichia coli* (8) |
| Other *streptococcus* spp. (7) | *Clostridium septicum* (1) | *Clostridium perfringens* (1) | *Pseudomonas aeruginosa* (6) |
| *Pseudomonas aeruginosa* (7) | *Citrobacter koseri* (1) | *Proteus mirabilis* (1) | *Enterococcus faecalis* (4) |
| *Enterococcus faecalis* (6) |  | *Pseudomonas aeruginosa* (1) | *Stenotrophomonas maltophilia* (4) |
| *Stenotrophomonas maltophilia* (5) |  | *Stenotrophomonas maltophilia* (1) | Other *streptococcus* spp. (3) |
| *Coagulase (-) Staphylococcus* (2) |  | Other *streptococcus* spp. (1) | *Coagulase (-) Staphylococcus* (2) |
| *Proteus mirabilis* (2) |  |  | *Serratia marcescens* (1) |
| *Enterobacter cloacae* (1) |  |  | *Proteus mirabilis* (1) |
| *Clostridium perfringens* (1) |  |  | *Ochrobactrum anthropic* (1) |
| *Clostridium septicum* (1) |  |  | *Klebsiella oxytoca* (1) |
| *Citrobacter koseri* (1) |  |  | *Enterobacter cloacae* (1) |
| *Citrobacter freundii* (1) |  |  | *Citrobacter freundii* (1) |
| *Bacteroides thetaiotaomicron* (1) |  |  | *Bacteroides thetaiotaomicron* (1) |
| *Enterococcus gallinarum* (1) |  |  | *Enterococcus gallinarum* (1) |
| *Klebsiella oxytoca* (1) |  |  |  |
| *Ochrobactrum anthropic* (1) |  |  |  |
| *Serratia marcescens* (1) |  |  |  |
|  |  |  |  |

**Additional Table S2. Isolated pathogen from Inje University Busan Paik Hospital**

| Overall (145) | Community-acquired (44) | Healthcare-associated (29) | Hospital-acquired (72) |
| --- | --- | --- | --- |
| *Escherichia coli* (28) | *Escherichia coli* (9) | *Klebsiella pneumonia* (7) | *Acinetobacter baumannii* (13) |
| *Klebsiella pneumonia* (25) | *Staphylococcus aureus* (9) | *Escherichia coli* (6) | *Escherichia coli* (13) |
| *Staphylococcus aureus* (17) | Other *streptococcus spp.* (9) | *Staphylococcus aureus* (3) | *Enterococcus faecium* (9) |
| *Acinetobacter baumannii* (14) | *Klebsiella pneumonia* (8) | *Pseudomonas aeruginosa* (3) | *Klebsiella pneumonia* (10) |
| Other *streptococcus* spp. (14) | *Proteus mirabilis* (2) | Other *streptococcus* spp. (3) | *Candida* spp. (8) |
| *Enterococcus faecium* (10) | *Streptococcus pneumoniae* (1) | *Coagulase (-) Staphylococcus* (1) | *Staphylococcus aureus* (5) |
| *Candida* spp. (8) | *Aeromonas hydrophila/caviae* (1) | *Streptococcus pneumoniae* (1) | *Pseudomonas aeruginosa* (3) |
| *Pseudomonas aeruginosa* (6) | *Klebsiella aerogenes* (1) | *Acinetobacter baumannii* (1) | Other *streptococcus* spp. (2) |
| *Proteus mirabilis* (3) | *Proteus penneri* (1) | *Bacteroides ovatus/xylanisolvens* (1) | *Stenotrophomonas maltophilia* (1) |
| *Coagulase (-) Staphylococcus* (2) | *Providencia rettgeri* (1) | *Enterobacter kobei* (1) | *Coagulase (-) Staphylococcus* (1) |
| *Streptococcus pneumoniae* (2) | *Salmonella group B* (1) | *Enterococcus faecium* (1) | *Proteus mirabilis* (1) |
| *Aeromonas hydrophila/caviae* (2) | *Vibrio vulnificus* (1) | *Haemophilus influenzae* (1) | *Citrobacter freundii* (1) |
| *Stenotrophomonas maltophilia* (1) |  |  | *Burkholderia cenocepacia* (1) |
| *Proteus penneri* (1) |  |  | *Acinetobacter ursingii* (1) |
| *Enterobacter kobei* (1) |  |  | *Aeromonas hydrophila/caviae* (1) |
| *Klebsiella aerogenes* (1) |  |  | *Corynebacterium striatum* (1) |
| *Haemophilus influenzae* (1) |  |  | *Cryptococcus neoformans* (1) |
| *Citrobacter freundii* (1) |  |  |  |
| *Acinetobacter ursingii* (1) |  |  |  |
| *Bacteroides ovatus/xylanisolvens* (1) |  |  |  |
| *Burkholderia cenocepacia* (1) |  |  |  |
| *Corynebacterium striatum* (1) |  |  |  |
| *Providencia rettgeri* (1) |  |  |  |
| *Salmonella group B* (1) |  |  |  |
| *Vibrio vulnificus* (1) |  |  |  |

**Additional Table S3. Age distribution of patients grouped by blood isolates (P=0.838)**

| Blood isolates | Age |
| --- | --- |
| *Klebsiella pneumonia* (54) | 73.58±9.25 |
| *Escherichia coli* (52) | 72.42±12.88 |
| *Staphylococcus aureus* (41) | 72.46±10.85 |
| *Acinetobacter baumannii* (34) | 69.90±10.88 |
| *Candida* spp. (29) | 66.81±14.85 |
| *Enterococcus faecium* (24) | 69.93±14.55 |
| Other *Streptococcus* spp*.* (21) | 73.00±10.91 |
| *Pseudomonas aeruginosa* (13) | 72.14±7.83 |

**Additional Table S4.** **Breakthrough bloodstream infection in patients with hospital-acquired bloodstream infection**

| Carbapenem single or plus non-glycopeptide | 22/184^a^ |
| --- | --- |
| Carbapenem plus glycopeptide | 15/156^b^ |
| Glycopeptide single or plus non-carbapenem | 4/184^c^ |

^a^ 8 *Acinetobacter baumannii*, 6 *Candida* spp*.*, 4 *Enterococcus faecium*, 2 *Enterococcus faecalis*, 2 *Staphylococcus aureus*, 2 *Stenotrophomonas maltophilia*, 1 *Pseudomonas aeruginosa*, and 1 *Staphylococcus haemolyticus*

^b^ 5 *Enterococcus faecium,* 4 *Acinetobacter baumannii*, 4 *Candida* spp*.*, 3 *Staphylococcus aureus*, 1 *Klebsiella pneumoniae*, and 1 *Corynebacterium striatum*

^c^ 2 *Acinetobacter baumannii*, 2 *Klebsiella pneumoniae*, 1 *Candida* spp*.*, and 1 *Escherichia coli*
